# (*E*)-*N*′-(4-Isopropyl­benzyl­idene)isonicotinohydrazide monohydrate

**DOI:** 10.1107/S1600536812009099

**Published:** 2012-03-10

**Authors:** Mashooq A. Bhat, Hatem A. Abdel-Aziz, Hazem A. Ghabbour, Madhukar Hemamalini, Hoong-Kun Fun

**Affiliations:** a Department of Pharmaceutical Chemistry, College of Pharmacy, King Saud University, PO Box 2457, Riyadh 11451, Saudi Arabia; b X-ray Crystallography Unit, School of Physics, Universiti Sains Malaysia, 11800 USM, Penang, Malaysia

## Abstract

In the title compound, C_16_H_17_N_3_O·H_2_O, the isonicotinohydrazide mol­ecule adopts an *E* conformation about the central C=N double bond. The dihedral angle between the pyridine and the benzene rings is 54.56 (15)°. In the crystal, mol­ecules are connected *via* N—H⋯O, O—H⋯N and O—H⋯O hydrogen bonds, forming a three-dimensional network.

## Related literature

For details and the biological activity of isoniazide, see: Bloom & Murray (1992 ▶); Loenhout-Rooyackers & Veen (1998 ▶); Hearn *et al.* (2009 ▶); Tripathi *et al.* (2011 ▶).
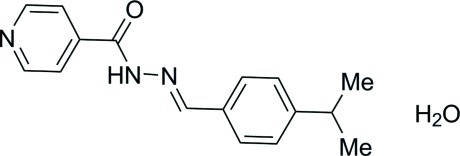


## Experimental

### 

#### Crystal data

C_16_H_17_N_3_O·H_2_O
*M* *_r_* = 285.34
Orthorhombic, 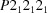

*a* = 7.7503 (2) Å
*b* = 11.7894 (3) Å
*c* = 17.2820 (4) Å
*V* = 1579.08 (7) Å^3^
*Z* = 4
Cu *K*α radiation
μ = 0.65 mm^−1^
*T* = 296 K
0.89 × 0.19 × 0.13 mm

#### Data collection

Bruker SMART APEXII CCD area-detector diffractometer
Absorption correction: multi-scan (*SADABS*; Bruker, 2009 ▶) *T*_min_ = 0.594, *T*_max_ = 0.920
6473 measured reflections
2939 independent reflections
2499 reflections with *I* > 2σ(*I*)
*R*_int_ = 0.032

#### Refinement

*R*[*F* ^2^ > 2σ(*F* ^2^)] = 0.049
*wR*(*F* ^2^) = 0.145
*S* = 1.04
2939 reflections
193 parameters
H-atom parameters constrained
Δρ_max_ = 0.22 e Å^−3^
Δρ_min_ = −0.28 e Å^−3^

### 

Data collection: *APEX2* (Bruker, 2009 ▶); cell refinement: *SAINT* (Bruker, 2009 ▶); data reduction: *SAINT*; program(s) used to solve structure: *SHELXTL* (Sheldrick, 2008 ▶); program(s) used to refine structure: *SHELXTL*; molecular graphics: *SHELXTL*; software used to prepare material for publication: *SHELXTL* and *PLATON* (Spek, 2009 ▶).

## Supplementary Material

Crystal structure: contains datablock(s) global, I. DOI: 10.1107/S1600536812009099/cv5248sup1.cif

Structure factors: contains datablock(s) I. DOI: 10.1107/S1600536812009099/cv5248Isup2.hkl

Supplementary material file. DOI: 10.1107/S1600536812009099/cv5248Isup3.cml

Additional supplementary materials:  crystallographic information; 3D view; checkCIF report

## Figures and Tables

**Table 1:** Hydrogen-bond geometry (Å, °)

*D*—H⋯*A*	*D*—H	H⋯*A*	*D*⋯*A*	*D*—H⋯*A*
N2—H1*N*1⋯O1*W*	0.85	1.90	2.757 (3)	176
O1*W*—H1*W*1⋯N1^i^	0.85	2.03	2.861 (3)	164
O1*W*—H2*W*1⋯O1^ii^	0.84	2.00	2.779 (3)	154

Symmetry codes: (i) 
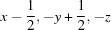
; (ii) 
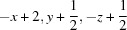
.

**Fig. 1.**
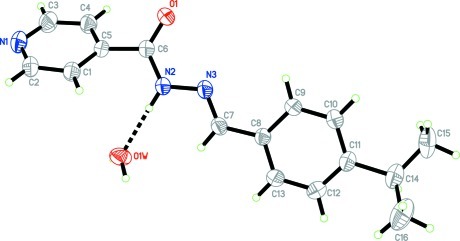
The asymmetric unit of the title compound, showing the atomic numbering and 30% probability displacement ellipsoids. Hydrogen bond is shown by dashed line.

**Fig. 2.**
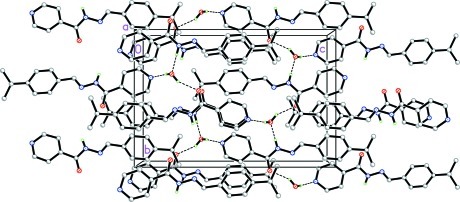
A portion of the crystal packing showing hydrogen bonds as dashed lines. H atoms not involved in hydrogen bonding were omitted for clarity.

## supplementary crystallographic information

### Comment

In the last decade, tuberculosis (TB) has reemerged as one of the leading causes of death in the world, reaching nearly three million deaths annually (Bloom & Murray, 1992). Therefore, the search for new drugs for tuberculosis is of the utmost importance. Treatment regimens are based on a long-term and combined chemotherapy. The most used first-choice drug is isoniazid, a bactericidal drug that acts both intracellularly in the macrophages and extracellularly in the necrotic tissue (Loenhout-Rooyackers & Veen, 1998). The derivatives of isoniazid have been found to possess potential tuberculostatic activity (Hearn *et al.*, 2009; Tripathi *et al.*, 2011). Herein, we present the crystal structure of the title compound, (I).

The asymmetric unit of (I) contains one N'-(4-isopropylbenzylidene) isonicotinohydrazide molecule and one water molecule (Fig. 1). The molecule adopts an *E* configuration about the central C7═N3 double bond. The dihedral angle between the pyridine (N1/C1–C5) and the benzene (C8–C13) rings is 54.56 (15)°. The hydrazine group is twisted slightly, with a C5-C6-N2-N3 torsion angle of -178.9 (2)°.

In the crystal, the molecules are connected *via* N—H···O, O—H···N and O—H···O hydrogen bonds (Table 1), forming a three-dimensional networks (Fig. 2).

### Experimental

The title compound was prepared by the reaction of 4-isopropyl benzaldehyde (0.15 g, 1 mmol) with isoniazid (0.14 g, 1 mmol) in EtOH (25 mL). After stirring for 3 h, at room temperature, the resulting mixture was concentrated. The precipitate was washed with EtOH to afford the title compound. Colourless blocks of the title compound suitable for X-ray structure determination were recrystallized from EtOH by the slow evaporation of the solvent at room temperature.

### Refinement

All hydrogen atoms were positioned geometrically [C–H = 0.93–0.98 Å; O–H = 0.84–0.85 Å] and were refined using a riding model, with *U*_iso_(H) = 1.2 or 1.5 *U*_eq_(C, O). A rotating group model was applied to the methyl groups Even though there is sufficient anomalous dispersion to find the absolute configuration as the compound crystallize out in a chiral space group and Cu radiation was used, this was unsuccessful as the crystal is a inversion twin [BASF ratio of 0.8 (4):0.2 (4)].

### Crystal data

**Table:**

C_16_H_17_N_3_O·H_2_O	*F*(000) = 608
*M**_r_* = 285.34	*D*_x_ = 1.200 Mg m^−^^3^
Orthorhombic, *P*2_1_2_1_2_1_	Cu *K*α radiation, λ = 1.54178 Å
Hall symbol: P 2ac 2ab	Cell parameters from 1751 reflections
*a* = 7.7503 (2) Å	θ = 4.5–65.6°
*b* = 11.7894 (3) Å	µ = 0.65 mm^−^^1^
*c* = 17.2820 (4) Å	*T* = 296 K
*V* = 1579.08 (7) Å^3^	Block, colourless
*Z* = 4	0.89 × 0.19 × 0.13 mm

### Data collection

**Table:**

Bruker SMART APEXII CCD area-detector diffractometer	2939 independent reflections
Radiation source: fine-focus sealed tube	2499 reflections with *I* > 2σ(*I*)
Graphite monochromator	*R*_int_ = 0.032
φ and ω scans	θ_max_ = 71.8°, θ_min_ = 4.5°
Absorption correction: multi-scan (*SADABS*; Bruker, 2009)	*h* = −9→8
*T*_min_ = 0.594, *T*_max_ = 0.920	*k* = −13→10
6473 measured reflections	*l* = −20→21

### Refinement

**Table:**

Refinement on *F*^2^	Hydrogen site location: inferred from neighbouring sites
Least-squares matrix: full	H-atom parameters constrained
*R*[*F*^2^ > 2σ(*F*^2^)] = 0.049	*w* = 1/[σ^2^(*F*_o_^2^) + (0.0715*P*)^2^ + 0.3351*P*] where *P* = (*F*_o_^2^ + 2*F*_c_^2^)/3
*wR*(*F*^2^) = 0.145	(Δ/σ)_max_ < 0.001
*S* = 1.04	Δρ_max_ = 0.22 e Å^−^^3^
2939 reflections	Δρ_min_ = −0.28 e Å^−^^3^
193 parameters	Extinction correction: *SHELXTL* (Sheldrick, 2008), Fc^*^=kFc[1+0.001xFc^2^λ^3^/sin(2θ)]^-1/4^
0 restraints	Extinction coefficient: 0.0045 (8)
Primary atom site location: structure-invariant direct methods	Absolute structure: Flack, H. D. (1983). *Acta Cryst.* A39, 876–881, 1072 Friedel pairs
Secondary atom site location: difference Fourier map	Flack parameter: 0.8 (4)

### Special details

**Table:**

Geometry. All s.u.'s (except the s.u. in the dihedral angle between two l.s. planes) are estimated using the full covariance matrix. The cell s.u.'s are taken into account individually in the estimation of s.u.'s in distances, angles and torsion angles; correlations between s.u.'s in cell parameters are only used when they are defined by crystal symmetry. An approximate (isotropic) treatment of cell s.u.'s is used for estimating s.u.'s involving l.s. planes.
Refinement. Refinement of F^2^ against ALL reflections. The weighted R-factor wR and goodness of fit S are based on F^2^, conventional R-factors R are based on F, with F set to zero for negative F^2^. The threshold expression of F^2^ > 2σ(F^2^) is used only for calculating R-factors(gt) etc. and is not relevant to the choice of reflections for refinement. R-factors based on F^2^ are statistically about twice as large as those based on F, and R- factors based on ALL data will be even larger.

### Fractional atomic coordinates and isotropic or equivalent isotropic displacement parameters (Å^2^)

**Table:**

	*x*	*y*	*z*	*U*_iso_*/*U*_eq_	
O1	1.1044 (3)	−0.05606 (15)	0.18186 (11)	0.0761 (6)	
N1	1.1036 (4)	0.1562 (2)	−0.06565 (14)	0.0839 (8)	
H1N1	0.9718	0.1817	0.2093	0.101*	
N2	1.0132 (3)	0.11714 (17)	0.22203 (11)	0.0573 (5)	
N3	1.0056 (3)	0.08469 (18)	0.29864 (11)	0.0578 (5)	
C1	1.1390 (4)	0.1925 (2)	0.06922 (16)	0.0666 (7)	
H1A	1.1742	0.2420	0.1080	0.080*	
C2	1.1526 (5)	0.2221 (3)	−0.00740 (18)	0.0785 (9)	
H2A	1.1990	0.2927	−0.0192	0.094*	
C3	1.0378 (5)	0.0560 (3)	−0.04701 (17)	0.0835 (9)	
H3A	1.0012	0.0089	−0.0869	0.100*	
C4	1.0204 (4)	0.0177 (2)	0.02743 (15)	0.0670 (7)	
H4A	0.9750	−0.0538	0.0374	0.080*	
C5	1.0715 (3)	0.0872 (2)	0.08717 (13)	0.0536 (5)	
C6	1.0648 (3)	0.0424 (2)	0.16813 (14)	0.0555 (6)	
C7	0.9297 (3)	0.1536 (2)	0.34324 (14)	0.0571 (6)	
H7A	0.8811	0.2185	0.3217	0.069*	
C8	0.9143 (3)	0.1369 (2)	0.42615 (13)	0.0515 (5)	
C9	0.9887 (4)	0.0448 (2)	0.46475 (15)	0.0604 (6)	
H9A	1.0450	−0.0113	0.4366	0.072*	
C10	0.9792 (4)	0.0366 (2)	0.54362 (15)	0.0689 (7)	
H10A	1.0299	−0.0253	0.5680	0.083*	
C11	0.8961 (4)	0.1178 (2)	0.58831 (15)	0.0692 (7)	
C12	0.8237 (4)	0.2085 (3)	0.54993 (16)	0.0699 (7)	
H12A	0.7684	0.2649	0.5783	0.084*	
C13	0.8312 (4)	0.2176 (2)	0.47065 (15)	0.0611 (6)	
H13A	0.7793	0.2792	0.4465	0.073*	
C14	0.8851 (9)	0.1105 (4)	0.67642 (19)	0.1318 (19)	
H14A	0.7599	0.1151	0.6836	0.158*	
C15	0.9192 (7)	−0.0015 (4)	0.7099 (2)	0.1196 (15)	
H15A	0.8882	−0.0011	0.7637	0.179*	
H15B	1.0396	−0.0190	0.7048	0.179*	
H15C	0.8521	−0.0576	0.6832	0.179*	
C16	0.9387 (9)	0.2120 (5)	0.7158 (2)	0.149 (2)	
H16A	0.8925	0.2121	0.7674	0.223*	
H16B	0.8968	0.2771	0.6883	0.223*	
H16C	1.0624	0.2145	0.7181	0.223*	
O1W	0.8651 (3)	0.32125 (15)	0.18150 (11)	0.0822 (7)	
H1W1	0.7748	0.3209	0.1531	0.123*	
H2W1	0.8803	0.3754	0.2127	0.123*	

### Atomic displacement parameters (Å^2^)

**Table:**

	*U*^11^	*U*^22^	*U*^33^	*U*^12^	*U*^13^	*U*^23^
O1	0.1132 (16)	0.0556 (10)	0.0594 (10)	0.0199 (10)	0.0095 (11)	0.0108 (9)
N1	0.114 (2)	0.0893 (18)	0.0484 (12)	0.0015 (16)	0.0134 (14)	0.0090 (12)
N2	0.0738 (13)	0.0546 (12)	0.0436 (10)	0.0047 (10)	0.0017 (9)	0.0057 (8)
N3	0.0726 (13)	0.0566 (12)	0.0443 (10)	0.0010 (10)	−0.0025 (9)	0.0063 (9)
C1	0.0849 (17)	0.0594 (15)	0.0555 (14)	−0.0062 (13)	0.0094 (13)	−0.0003 (12)
C2	0.107 (2)	0.0670 (18)	0.0617 (17)	−0.0041 (16)	0.0213 (16)	0.0106 (14)
C3	0.113 (3)	0.086 (2)	0.0525 (15)	−0.0043 (19)	−0.0001 (16)	−0.0066 (14)
C4	0.0819 (18)	0.0642 (16)	0.0547 (14)	−0.0060 (14)	0.0020 (13)	0.0000 (12)
C5	0.0623 (13)	0.0537 (13)	0.0448 (11)	0.0029 (11)	0.0079 (10)	0.0043 (10)
C6	0.0638 (13)	0.0541 (14)	0.0486 (12)	0.0022 (11)	0.0030 (11)	0.0030 (10)
C7	0.0697 (14)	0.0512 (13)	0.0504 (13)	0.0021 (12)	−0.0057 (11)	0.0055 (10)
C8	0.0559 (11)	0.0526 (13)	0.0462 (11)	0.0002 (10)	−0.0027 (10)	0.0009 (9)
C9	0.0750 (16)	0.0530 (13)	0.0531 (13)	0.0117 (12)	0.0005 (12)	0.0027 (10)
C10	0.0871 (19)	0.0657 (16)	0.0538 (14)	0.0139 (14)	−0.0047 (13)	0.0083 (12)
C11	0.0913 (19)	0.0708 (17)	0.0455 (13)	0.0007 (15)	0.0039 (13)	−0.0005 (12)
C12	0.0798 (18)	0.0674 (16)	0.0624 (16)	0.0095 (14)	0.0115 (13)	−0.0081 (14)
C13	0.0659 (15)	0.0567 (14)	0.0607 (14)	0.0118 (12)	−0.0004 (12)	0.0022 (11)
C14	0.238 (6)	0.108 (3)	0.0494 (18)	0.018 (4)	0.006 (3)	0.0001 (19)
C15	0.149 (4)	0.149 (4)	0.0615 (19)	−0.022 (3)	0.008 (2)	0.034 (2)
C16	0.204 (5)	0.180 (5)	0.062 (2)	−0.002 (5)	−0.027 (3)	−0.026 (3)
O1W	0.1251 (18)	0.0586 (11)	0.0628 (11)	0.0163 (11)	−0.0286 (12)	−0.0119 (9)

### Geometric parameters (Å, º)

**Table:**

O1—C6	1.224 (3)	C9—C10	1.368 (3)
N1—C3	1.326 (4)	C9—H9A	0.9300
N1—C2	1.327 (4)	C10—C11	1.389 (4)
N2—C6	1.343 (3)	C10—H10A	0.9300
N2—N3	1.379 (3)	C11—C12	1.377 (4)
N2—H1N1	0.8550	C11—C14	1.528 (4)
N3—C7	1.266 (3)	C12—C13	1.376 (4)
C1—C2	1.373 (4)	C12—H12A	0.9300
C1—C5	1.383 (4)	C13—H13A	0.9300
C1—H1A	0.9300	C14—C16	1.438 (6)
C2—H2A	0.9300	C14—C15	1.465 (6)
C3—C4	1.370 (4)	C14—H14A	0.9800
C3—H3A	0.9300	C15—H15A	0.9600
C4—C5	1.376 (4)	C15—H15B	0.9600
C4—H4A	0.9300	C15—H15C	0.9600
C5—C6	1.496 (3)	C16—H16A	0.9600
C7—C8	1.451 (3)	C16—H16B	0.9600
C7—H7A	0.9300	C16—H16C	0.9600
C8—C13	1.382 (3)	O1W—H1W1	0.8541
C8—C9	1.398 (3)	O1W—H2W1	0.8437
			
C3—N1—C2	116.6 (3)	C9—C10—C11	122.0 (3)
C6—N2—N3	119.78 (19)	C9—C10—H10A	119.0
C6—N2—H1N1	121.2	C11—C10—H10A	119.0
N3—N2—H1N1	118.5	C12—C11—C10	117.1 (2)
C7—N3—N2	115.2 (2)	C12—C11—C14	120.1 (3)
C2—C1—C5	118.3 (3)	C10—C11—C14	122.7 (3)
C2—C1—H1A	120.9	C13—C12—C11	121.5 (3)
C5—C1—H1A	120.9	C13—C12—H12A	119.2
N1—C2—C1	124.1 (3)	C11—C12—H12A	119.2
N1—C2—H2A	117.9	C12—C13—C8	121.4 (3)
C1—C2—H2A	117.9	C12—C13—H13A	119.3
N1—C3—C4	124.0 (3)	C8—C13—H13A	119.3
N1—C3—H3A	118.0	C16—C14—C15	120.7 (4)
C4—C3—H3A	118.0	C16—C14—C11	114.1 (4)
C3—C4—C5	118.7 (3)	C15—C14—C11	115.8 (3)
C3—C4—H4A	120.7	C16—C14—H14A	100.3
C5—C4—H4A	120.7	C15—C14—H14A	100.3
C4—C5—C1	118.4 (2)	C11—C14—H14A	100.3
C4—C5—C6	118.8 (2)	C14—C15—H15A	109.5
C1—C5—C6	122.6 (2)	C14—C15—H15B	109.5
O1—C6—N2	124.2 (2)	H15A—C15—H15B	109.5
O1—C6—C5	120.5 (2)	C14—C15—H15C	109.5
N2—C6—C5	115.3 (2)	H15A—C15—H15C	109.5
N3—C7—C8	123.5 (2)	H15B—C15—H15C	109.5
N3—C7—H7A	118.2	C14—C16—H16A	109.5
C8—C7—H7A	118.2	C14—C16—H16B	109.5
C13—C8—C9	117.4 (2)	H16A—C16—H16B	109.5
C13—C8—C7	119.6 (2)	C14—C16—H16C	109.5
C9—C8—C7	122.9 (2)	H16A—C16—H16C	109.5
C10—C9—C8	120.5 (2)	H16B—C16—H16C	109.5
C10—C9—H9A	119.7	H1W1—O1W—H2W1	119.0
C8—C9—H9A	119.7		
			
C6—N2—N3—C7	−169.0 (3)	N3—C7—C8—C13	179.0 (3)
C3—N1—C2—C1	0.0 (5)	N3—C7—C8—C9	2.7 (4)
C5—C1—C2—N1	0.6 (5)	C13—C8—C9—C10	−0.3 (4)
C2—N1—C3—C4	−0.9 (5)	C7—C8—C9—C10	176.0 (3)
N1—C3—C4—C5	1.0 (5)	C8—C9—C10—C11	0.2 (5)
C3—C4—C5—C1	−0.3 (4)	C9—C10—C11—C12	−0.4 (5)
C3—C4—C5—C6	−175.6 (3)	C9—C10—C11—C14	−179.6 (4)
C2—C1—C5—C4	−0.4 (4)	C10—C11—C12—C13	0.8 (5)
C2—C1—C5—C6	174.7 (3)	C14—C11—C12—C13	−179.9 (4)
N3—N2—C6—O1	1.3 (4)	C11—C12—C13—C8	−1.1 (5)
N3—N2—C6—C5	−178.9 (2)	C9—C8—C13—C12	0.8 (4)
C4—C5—C6—O1	38.5 (4)	C7—C8—C13—C12	−175.7 (3)
C1—C5—C6—O1	−136.6 (3)	C12—C11—C14—C16	−50.3 (7)
C4—C5—C6—N2	−141.4 (3)	C10—C11—C14—C16	128.9 (5)
C1—C5—C6—N2	43.6 (4)	C12—C11—C14—C15	162.9 (4)
N2—N3—C7—C8	−177.5 (2)	C10—C11—C14—C15	−17.9 (7)

### Hydrogen-bond geometry (Å, º)

**Table:**

*D*—H···*A*	*D*—H	H···*A*	*D*···*A*	*D*—H···*A*
N2—H1*N*1···O1*W*	0.85	1.90	2.757 (3)	176
O1*W*—H1*W*1···N1^i^	0.85	2.03	2.861 (3)	164
O1*W*—H2*W*1···O1^ii^	0.84	2.00	2.779 (3)	154

Symmetry codes: (i) *x*−1/2, −*y*+1/2, −*z*; (ii) −*x*+2, *y*+1/2, −*z*+1/2.
